# The Intra- and Inter-Rater Reliability of a Hip Rotation Range-of-Motion Measurement Using a Smartphone Application in Academy Football (Soccer) Players

**DOI:** 10.3390/sports9110148

**Published:** 2021-10-26

**Authors:** Paul Spork, James O’Brien, Morris Sepoetro, Maximilian Plachel, Thomas Stöggl

**Affiliations:** 1 Physiotherapy, Salzburg University of Applied Sciences, Urstein Süd 1, 5412 Puch, Austria; 2 Red Bull Athlete Performance Center, Brunnbachweg 71, 5303 Salzburg, Austria; drjamesob@outlook.com (J.O.); morris.sepoetro@gmail.com (M.S.); max.plachel@hotmail.com (M.P.); thomas.stoeggl@sbg.ac.at (T.S.); 3 The Australian Centre for Research into Injury in Sport and Its Prevention (ACRISP), Edith Cowan University, 270 Joondalup Drive, Joondalup, WA 6027, Australia; 4 Department of Sport and Exercise Science, University of Salzburg, Schlossallee 49, 5400 Hallein, Austria

**Keywords:** sports, prevention, groin

## Abstract

The clinical assessment of hip rotation range-of-motion (ROM) is important for managing hip and groin injuries in footballers. Previously published reliability studies on hip ROM have employed protocols that are difficult to replicate under everyday clinical conditions. This single trial, intra- and inter-rater reliability study included 41 male academy football (soccer) players, aged 14–15 years, from one European football academy. Passive hip internal rotation (IR) and external rotation (ER) ROM were measured in supine with hip and knee flexed to 90°. The ROM was determined using a smartphone application, with the smartphone attached to the lower leg. The tests were performed on two separate occasions, one week apart, by two different physiotherapists and on both sides (left and right hips). Reliability was evaluated using Intra-Class Correlation Coefficients (ICCs) and Minimal Detectable Change (MDC). Hip IR and ER ROM displayed moderate to good intra-rater agreement (ICCs 0.54–0.75), with MDCs ranging from 10.9° to 16.4°. Inter-rater reliability displayed poor to moderate reliability (ICCs 0.33–0.75), with MDCs ranging from 11.7° to 16.5°. A hip rotation ROM test using a smartphone application and a protocol closely reflecting everyday clinical conditions displayed moderate to good intra-rater reliability and poor to moderate inter-rater reliability. Due to the high MDCs, the practical applicability of this test procedure is limited and further refinement is necessary.

## 1. Introduction

Hip and groin injuries are common in football, accounting for 14% of all injuries in professional teams [1] and up to 33% of all injuries in elite youth teams [2]. These injuries also have a high recurrence rate, [3] making them an important focus of preventive and rehabilitative efforts. Decreased hip rotation range of motion (ROM) is associated with hip and groin injuries in athletes [4,5,6,7], and assessing hip rotation ROM is an important clinical test [8]. Accordingly, developing reliable and practical hip rotation ROM tests is important to support the management of hip and groin injuries in football and other sport settings.

Hip rotation ROM is commonly assessed using a goniometer [9,10,11,12] or inclinometer [8,13,14]. In addition to measuring hip internal rotation (IR) and hip external rotation (ER), total rotation (TR) is often calculated by adding IR and ER [4]. In previous reliability studies employing a goniometer, intra-rater agreement for hip rotation ROM ranged from moderate to excellent [9,10,12], while the inter-rater agreement was poor to good [9,10,11,12]. Studies employing an inclinometer to measure hip rotation ROM have reported higher levels of reliability compared to goniometers; inter-rater values ranged from moderate to excellent for IR and moderate to good for ER, while intra-rater values ranged from good to excellent for IR and moderate to excellent for ER [8,13,14]. Hip rotation can be measured in different positions with the subject either sitting or in supine and with the hip joint positioned either in neutral or 90° flexion. The supine, 90° hip flexion position is the most commonly reported test position [8] and displays higher reliability than tests in sitting [9]. The reported minimal detectable change (MDC) values in the supine, 90° hip flexion position were 7.9° for IR and 7.5° for ER [9].

Technological advances in optical and inertial motion capture have presented new alternatives for measuring joint ROM. Three-dimensional motion capture is commonly recognized as the gold standard, but its cost and the required expertise limit its clinical application. [15] Wearable inertial measurement units (IMUs) present a practical, mobile, and low-cost alternative, which have been employed in the clinical assessment of the shoulder [16], knee [17,18], elbow [19], and spine [20,21,22]. The integration of inertial sensors into smartphones, along with the development of specific software applications, now allows clinicians to use smartphones to measure joint ROM [23,24]. The use of smartphones has a number of potential advantages, including high accessibility, ease of use, and relatively low cost. However, the reliability and validity of smartphone applications to measure joint ROM has been questioned [23].

Recently, a number of studies focusing on the reliability of goniometric smartphone applications have emerged [25]. Although studies specific to the hip joint remain rare [23], two recent studies assessed hip rotation measurements in healthy adults. Charlton et al. [13] reported intra-rater reliability values ranging from moderate to excellent for IR and ER hip rotation in a seated and supine (0° hip flexion) position, while St-Pierre et al. [26] found good intra-rater reliability and moderate to good inter-rater reliability for hip IR in the supine (90° hip flexion) position. However, the reported MDC values were high, ranging up to 19.1° for intra-tester and 22.6° for inter-tester reliability [26].

For clinicians applying the findings of these published reliability studies to everyday clinical practice, it is important to carefully consider the specific study population, sample size, and test procedure employed in studies [27]. Most reliability studies on hip ROM employed small samples [9,12,26], focused on healthy adults with injuries as an exclusion criterion [9,13,26,28], and performed multiple test trials [13,28]. Some studies also employ two testers and additional equipment to fixate the pelvis [12]. While such measures are likely to improve reliability values, they are time-intensive and difficult to replicate under everyday clinical conditions, where clinicians often have limited time and resources [29].

In professional sports settings, clinical tests will often be performed under time pressure, before training sessions (to decide if the player can participate), and in the absence of standardized warm-ups [29,30]. Hip rotation ROM tests are also commonly included in test batteries [10], involving a range of musculoskeletal tests being performed on a group of players in a limited time. In such situations, the use of multiple trials is unpractical. Another important factor is the exclusion of participants with previous hip and groin pain in published reliability studies, which contrasts with the situation in most football clubs, where hip and groin pain are common [1,2]. Further potential limitations of previous studies are lack of sample size calculation and blinding of the testers, high MDC values, along with a lack of testing for normality and variance homogeneity, which are statistical assumptions for calculating reliability with Intraclass Correlation Coefficients (ICC).

To address these limitations, our study aimed to evaluate the reliability of a hip rotation ROM test, using a smartphone application and a test procedure closely reflecting everyday practice in the football academy soccer setting (e.g., single trial, one tester, and no warm-up). We hypothesized that excellent reliability (ICC > 0.90) and low Minimal Detectable Change (MDC) values (<10°) could be achieved. The findings of this study can directly inform clinicians employing these tests under real-world clinical settings for the diagnosis, rehabilitation, and prevention of hip and groin injuries.

## 2. Materials and Methods

The participants were male football players from two football teams (Under-15 and Under-16) in one European football academy. The teams competed in the highest national youth competition and typically played one game per week, in addition to 6–7 training sessions. The players routinely took part in a bi-annual musculoskeletal screening test battery, including the hip rotation ROM test investigated in this study. All players presenting to the routine screening test in December 2020 were eligible for participation. Current injuries, including hip/groin pain, were not exclusion criteria unless the team’s medical staff judged the injury to be a contraindication for hip ROM testing. A-priori sample size calculations, based on the methods of Walter et al. [31] were conducted; assuming a significance level (α) = 0.05, probability of type II error (β) = 0.2, acceptable reliability (ρ0) = 0.5, and expected reliability (ρ1) = 0.8, a sample size of 22 players was necessary. However, to allow for potential dropouts due to injury or illness (including COVID) considerably more participants were recruited. This study was approved by the Human Ethics Committee of the Paris Lodron University Salzburg, Austria (GZ 60/2020). Written informed consent forms were completed by all participants or their parents/legal guardians.

Passive hip IR and ER ROM were measured in two separate testing sessions, separated by seven days. On each occasion, the players were tested on both the left and right hips by two different testers (both physiotherapists with four years of experience). The testers were in separate rooms, and the order in which players were tested in each session (physiotherapist 1 and physiotherapist 2) was randomized, using block randomization due to the different school timetables of the players. Additionally, both the testers and participants were blinded to the hip rotation ROM results, with an assistant recording the values. In order to replicate normal conditions for testing hip rotation ROM in the academy, the sessions were conducted with a single measure prior to training with no warm-up.

**Figure 1:**
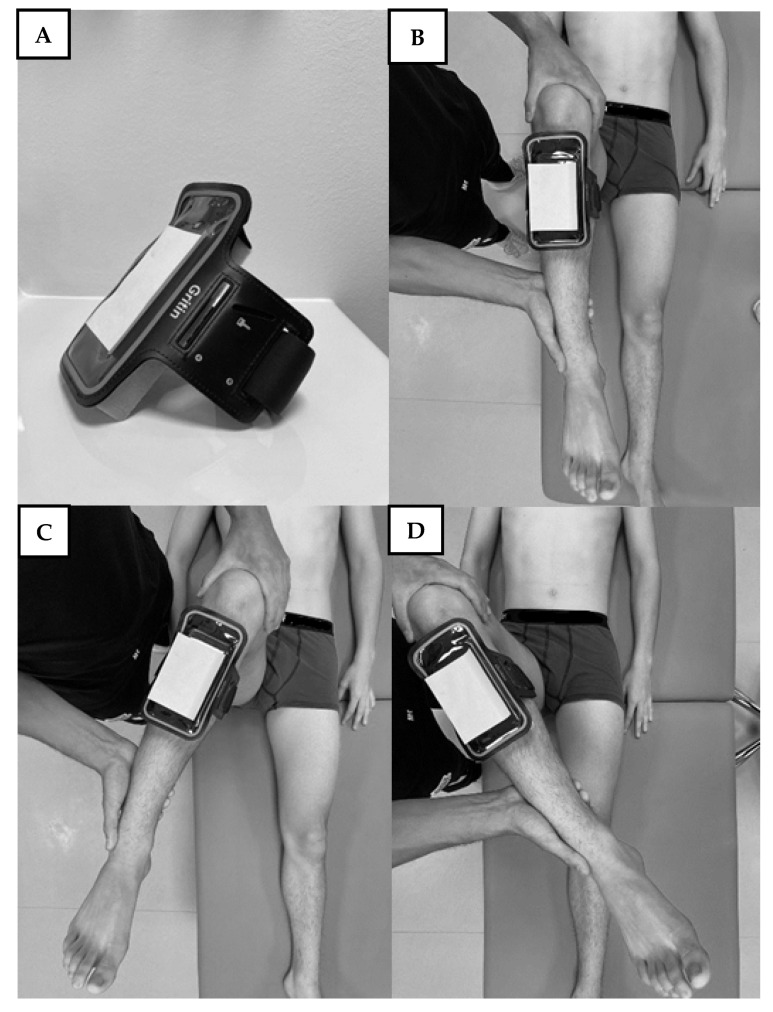
Test procedure for measuring hip rotation range-of-motion (ROM). (**A**): Smartphone running armband and smartphone (display concealed for blinding), (**B**): Starting position, (**C**): Internal Rotation, (**D**): External rotation.

The test procedure used in this study was similar to a previous report by St-Pierre et al. [26]. The participant was lying supine on a plinth, towards the side of the hip to be tested. The left side was always tested before the right. A running armband (Gritin G3223, Irvinestown, UK, Figure 1A) with additional foam padding, was used to attach the smartphone to the player’s lower leg. Two identical iPhones (iPhone 8, iOS 13.7, Apple, Los Altos, CA, USA) and the application yROM (Version 1.7.1, Healthcare Technologies LLC, Norman Park, GA, USA) were used. The player’s hip and knee were positioned in 90° flexion [8,10,28] with the lower leg parallel to the long axis of the plinth and the thigh perpendicular to the plinth (Figure 1B). This was set as the starting position (0°) in the smartphone application. The hip was then passively moved into IR (Figure 1C). The end-of-range position was defined as the point at which either firm resistance was reached, or compensatory movement was noted at the pelvis or trunk. To blind the examiner (and the participants) from the values displayed on the mobile phone, the screen was covered with adhesive paper, 100 mm × 75 mm (Figure 1D). When the end-of-range position had been reached (as determined by the tester), the assistant tapped the mobile phone display to record the measurement and entered the values into a Microsoft Excel^TM^ (Microsoft Corporation, Redmond, WA, USA)document. The same procedure was used to test hip ER (Figure 1D), before repeating both movements on the right hip. For hip ER, the end-of-range position was defined as the point at which either firm resistance was reached, or compensatory movement was noted at the contralateral foot/heel.

Prior to the study, the two testers took part in two, one-hour training sessions to familiarize themselves with the study procedure. In the first training session, the testers practiced the procedure on adult work colleagues in the academy, while the second training session was performed on academy players who were not involved in the study.

### Statistical Analysis

The raw data were entered into Microsoft Excel^TM^, and TR was calculated as the sum of IR and ER, before entering the data into SPSS^TM^ (IBM, Version 27.0, Armonk, NY, USA) for analysis. The normal distribution (Shapiro-Wilk test) and the variance homogeneity (Levene’s test) were calculated. For all data satisfying statistical assumptions (variation and homogeneity), Intraclass Correlation Coefficients (ICC), Standard Error of Measurement (SEM), and Minimal Detectable Change (MDC) were calculated. For ICC, two-way mixed models with absolute agreement (3, 1) were used. Descriptive statistics and ICC values were calculated in SPSS, while SEM and MDC were calculated in Excel, using the equations of Weir (SEM = SD × √ (1 − ICC) and MDC = SEM × 1.96 × √2) [32]. The Coefficient of Variation (CV) was calculated in Excel for all data, using the equation CV = (Standard Deviation/Mean) × 100. For data not satisfying statistical assumptions for ICCs, Spearman’s rank correlation coefficient was calculated. Inter- and intra-rater reliability was calculated between the two testing sessions (seven days apart) as this reflected the normal use of this test for monitoring selected players in the soccer academy. The ICC values were interpreted as follows: <0.50 = poor, 0.50–0.75 = moderate, 0.75–0.90 = good and >0.90 = excellent [33]. Spearman’s rank correlation coefficient was interpreted as: <0.10 negligible correlation, 0.10–0.39 weak correlation, 0.40–0.69, moderate correlation, 0.70–0.89 strong correlation, >0.90 very strong correlation [34].

## 3. Results

A total of 41 players (82 hips) were tested in the first session. For the second session, there were 11 dropouts: three due to injury, one due to illness, and seven due to COVID quarantine measures. Accordingly, 30 players (60 hips) were included in the final analysis. The data for IR and ER of the right hip were normally distributed (*p* > 0.05) for both sessions and testers. For the left hip, all data with the exception of ER from one tester in the first session were normally distributed (*p* > 0.05). The TR of the left hip by tester one was normally distributed in both sessions, as was the TR for the right hip of tester two in the first session (*p* > 0.05). There was no significant difference in the variance homogeneity for all normally distributed data (*p* > 0.05). Descriptive statistics for hip rotation measurements are presented in Table 1.

The mean IR hip ROM of the participants ranged from 40.4° to 43.5° (SD 7.2° to 7.9°), while the mean ER hip ROM ranged from 58.4° to 60.7° (SD 7.0° to 8.8°) and the mean TR hip ROM ranged from 98.9° to 104° (10.6° to 12.9°). The ICCs, SEMs, MDCs, and CVs for intra-rater reliability of hip rotation measurements are displayed in Table 2. The ICCs for the intra-rater reliability of IR, ER, and TR indicated moderate to good agreement (0.54–0.75). Spearman’s rank correlation coefficients (r_s_) indicated moderate agreement (0.47–0.67). The ICCs, SEMs, MDCs, and CVs for inter-rater reliability of hip rotation measurements are displayed in Table 3. The inter-rater reliability values ranged from poor to moderate (ICCs 0.33–0.75). Spearman’s rank correlation coefficients indicated moderate agreement (0.41–0.55).

## 4. Discussion

Hip and groin injuries are a major issue in elite football [1,2], and assessing hip rotation ROM is an important part of the clinical examination of these injuries [4,7,8]. Although a number of previous studies have investigated the reliability of hip rotation ROM, very few have employed smartphone technology and the test protocols of previous studies have not reflected real-world clinical conditions. This study is the first to investigate the reliability of hip rotation ROM utilising smartphone technology and a protocol closely reflecting everyday clinical practice. Hip rotation ROM measures displayed moderate to good intra-rater agreement and poor to moderate inter-rater reliability.

Direct comparison of the study findings to previously reported studies is difficult due to differences in study populations and protocols. Charlton et al. [13] found moderate to excellent intra-rater reliability (ICCs 0.63–0.94) for IR and ER compared to the moderate to good agreement (ICCs 0.54–0.75) in our study. However, Charlton et al. [13] investigated a different study population (healthy adults), employed different hip ROM test positions, and calculated reliability based on the average of three trials. St. Pierre et al. [26] reported good intra-rater (ICC 0.70–0.90) and moderate to good inter-rater (ICC 0.65–0.83) reliability for hip IR, using a single-trial measurement protocol. These ICC values are also somewhat higher than in the current study, possibly due to the different study populations; St Pierre et al. [26] studied symptom-free adults with low levels of sport participation, compared to the younger, academy football players in our study. Additionally, the lack of blinding of testers may have impacted the study findings. Following the completion of our study, a further study [28] using smartphone technology reported good to excellent intra-rater reliability (ICCs 0.85–0.97) and fair to good inter-rater reliability (ICCs 0.43–0.90) for hip rotation measures, in a sample of 24 healthy adults tested in a laboratory setting.

Taken together, the above research findings suggest that hip rotation ROM can be measured with a high level of reliability on healthy subjects under controlled conditions [13,26,28]. This is particularly true for the intra-rater reliability of IR measures [13,26,28]. However, the findings of our study suggest that the reliability of hip rotation ROM measures under everyday clinical conditions is poor to moderate. This represents an important limitation towards the widespread use of this measurement technique under real-world conditions, particularly when multiple testers assess players.

The MDC values can assist clinicians in interpreting hip rotation ROM measurements, with values beyond the MDC indicating, with 95% probability, an actual change as opposed to measurement error. For intra-rater reliability, MDCs in our study ranged from 10.9° and 13.4° for hip IR, slightly lower than the values (range 12.6° to 19.1°) reported by St. Pierre et al. [26]. In our opinion, these MDCs are large and limit the practical application of the hip rotation ROM tests used in this study. For example, if using this test procedure for weekly monitoring of hip IR in a football academy, a change of more than 13.4° would be required to be confident that a real change in IR was being observed, as opposed to measurement error alone. This is a substantial change when considered as a proportion of the hip IR ROM found in this study (e.g., for tester 1 and the left hip, the mean IR was 41.4°). The MDCs are also considerably higher than reported improvements in hip rotation ROM following interventions [35,36]. For example, a recent randomized controlled trial investigating the effect of manual therapy on hip ROM, reported a 7.6° improvement in mean hip IR and 5.6° improvement in mean ER, following three sessions of passive hip joint mobilization [35]. In order to effectively assess hip rotation ROM, across different sessions and different testers, a higher level of inter- and intra-rater reliability is desirable.

A secondary outcome of this study is the normative data for hip rotation ROM in male academy football players. The mean IR values (40.4° to 43.5° across testers and left/right hips) and ER values (58.7° to 60.7°) align with the findings of one previous study in ten academy athletes, 15.3 ± 1.6 years old, four of whom were football players [37]. Another study found lower IR values (mean 35.9°) and ER values (mean 49°) in academy football players [10], possibly reflecting the different ages (12–20 years old), ethnicity, or maturation of the participants. These normative data can inform clinicians working with academy football players in this specific age range.

A strength of this study was the large sample size with 41 participants compared to 20–24 participants in previous studies [13,26,28]. Additionally, both hips (total 82) were tested. Further strengths were the blinding and randomized order of testers. The reliability of the test protocol we used could potentially be improved by performing a standardized warm-up, using multiple trials, and employing two testers rather than one. However, these additional measures would make the test procedure longer and less practical in the academy football setting. More extensive familiarization of the testers and the use of tight-fitting smartphone cases [28] may also improve reliability. The testers reported noticing a degree of movement of the smartphone within the smartphone running armband on some occasions. A potential limitation of this study was the timing of test sessions. Both sessions were performed after school and before the soccer training sessions, but testing at the exact same time of the day was not possible due to short-term scheduling changes in the football academy. There was a considerable drop-out rate for the second session, in part due to COVID-related quarantine measures. The order of testing (left and right side) was not randomized, which reflected the normal test procedure in the football academy but may have influenced the findings.

As two smartphones were used, angular position differences between the two inertial sensors may have been a source of error [38]. To our knowledge, the specific application employed in this study has not been validated. Furthermore, the accuracy of smartphone IMUs may not be comparable to research-grade IMUs [39]. Future studies should consider increased familiarization of the testers before data collection, the use of tightly fitting smartphone running armbands, and comparing the reliability of single-trial tests with multiple-trial tests.

## 5. Conclusions

A supine hip rotation ROM measurement, using a smartphone application, demonstrated moderate to good intra-rater reliability and poor to moderate inter-rater reliability. The MDCs for IR and ER ROM ranged from 10.9°–16.4° for measurements by the same tester and 11.7°–16.5° for measurements between testers. The test protocol in this study closely simulated normal clinical conditions, but the high MDC values limit its practical application. Employing a standardized warm-up, extensive training of testers, and improvement of the smartphone fixation to the leg might improve reliability values, but this remains to be proven.

## Figures and Tables

**Table 1:** Descriptive statistics for hip rotation measurements.

	Tester 1				Tester 2			
	Left Hip		Right Hip		Left Hip		Right Hip	
	Mean	SD	Mean	SD	Mean	SD	Mean	SD
IR	41.4°	7.9°	40.5°	7.2°	40.4°	7.6°	43.5°	7.4°
ER	58.7°	8.3°	58.4°	8.8°	60.7°	7.0°	60.6°	7.8°
TR	100.0°	12.9°	98.9°	12.6°	101.1°	11.0°	104.0°	10.6°

SD = standard deviation, IR = internal rotation, ER = external rotation, TR = total rotation.

**Table 2:** Intra-rater reliability for hip internal rotation and external rotation.

	Tester 1								Tester 2							
	Left Hip				Right Hip				Left Hip				Right Hip			
	ICC	SEM	MDC	CV	ICC/r_s_	SEM	MDC	CV	ICC/rₛ	SEM	MDC	CV	ICC/r_s_	SEM	MDC	CV
IR	0.75	3.9°	10.9°	6.0%	0.59	4.6°	12.7°	6.1%	0.60	4.8°	13.4°	7.2%	0.64	4.4°	12.2°	6.0%
ER	0.59	5.4°	14.8°	4.6%	0.54	5.9°	16.4°	5.5%	0.51 *	*	*	5.5%	0.73	4.0°	11.2°	3.8%
TR	0.71	7.0°	19.3°	3.7%	0.47 *	*	*	4.3%	0.49 *	*	*	5.0%	0.67 *	*	*	3.4%

* For data not satisfying statistical assumptions for ICCs, Spearman’s rank correlation coefficients (r_s_) were calculated and SEM/MDC were omitted. ICC = Intraclass correlation coefficient, SEM = standard error of measurement, MDC = minimal detectable change, CV = Coefficient of Variation, IR = internal rotation, ER = external rotation, TR = total rotation.

**Table 3:** Inter-rater reliability for hip internal rotation and external rotation.

	Tester 1 to Tester 2 ^†^								Tester 2 to Tester 1 ^†^							
	Left Hip				Right Hip				Left Hip				Right Hip			
	ICC/r_s_	SEM	MDC	CV	ICC/rₛ	SEM	MDC	CV	ICC/r_s_	SEM	MDC	CV	ICC/rₛ	SEM	MDC	CV
IR	0.57	5.2	14.4	7.2%	0.33	5.9	16.5	8.5%	0.64	4.6	12.8	6.7%	0.54	5.0	13.7	6.9%
ER	0.48	5.5	15.2	6.0%	0.57	5.4	14.9	5.3%	0.48 *	*	*	4.6%	0.75	4.2	11.7	4.1%
TR	0.43 *	*	*	5.1%	0.41 *	*	*	5.6%	0.44 *	*	*	3.9%	0.55 *	*	*	3.5%

* For data not satisfying statistical assumptions for ICCs, Spearman’s rank correlation coefficients (r_s_) were calculated and SEM/MDC were omitted. ^†^ Inter-rater reliability was calcaulated twice: first comparing Tester 1 in the first session with Tester 2 in the second session and then vice versa. ICC = Intraclass Correlation Coefficient, SEM = standard error of measurement, MDC = minimal detectable change, CV = Coefficient of Variation, IR = internal rotation, ER = external rotation, TR = total rotation.

## Data Availability

The data is not yet publicly available.
